# Kismet Positively Regulates Glutamate Receptor Localization and Synaptic Transmission at the *Drosophila* Neuromuscular Junction

**DOI:** 10.1371/journal.pone.0113494

**Published:** 2014-11-20

**Authors:** Rupa Ghosh, Srikar Vegesna, Ramia Safi, Hong Bao, Bing Zhang, Daniel R. Marenda, Faith L. W. Liebl

**Affiliations:** 1 Department of Biology, Drexel University, Philadelphia, Pennsylvania, United States of America; 2 Division of Biological Sciences, University of Missouri, Columbia, Missouri, United States of America; 3 Department of Neurobiology and Anatomy, Drexel University College of Medicine, Philadelphia, Pennsylvania, United States of America; 4 Department of Biological Sciences, Southern Illinois University Edwardsville, Edwardsville, Illinois, United States of America; Columbia University, United States of America

## Abstract

The *Drosophila* neuromuscular junction (NMJ) is a glutamatergic synapse that is structurally and functionally similar to mammalian glutamatergic synapses. These synapses can, as a result of changes in activity, alter the strength of their connections via processes that require chromatin remodeling and changes in gene expression. The chromodomain helicase DNA binding (CHD) protein, Kismet (Kis), is expressed in both motor neuron nuclei and postsynaptic muscle nuclei of the *Drosophila* larvae. Here, we show that Kis is important for motor neuron synaptic morphology, the localization and clustering of postsynaptic glutamate receptors, larval motor behavior, and synaptic transmission. Our data suggest that Kis is part of the machinery that modulates the development and function of the NMJ. Kis is the homolog to human CHD7, which is mutated in CHARGE syndrome. Thus, our data suggest novel avenues of investigation for synaptic defects associated with CHARGE syndrome.

## Introduction

Synapses in the nervous system must be stable yet labile to retain existing memories and help form new memories. Several synaptic signaling molecules are important for synapse formation and/or maintenance including Wnts [1], [2], bone morphogenetic proteins (BMPs) [3], and neurotrophins [4] amongst others. These signaling molecules alter intracellular signaling and target cell transcription to convert transient cellular changes to stable, functional alterations [5] that ultimately maintain long-term synaptic connections and synaptic activity. Transcriptional activation is required for long-term potentiation (LTP) [6], [7], which is characterized by enhanced synaptic transmission as a result of increased synaptic activity, and learning and memory [8]. Epigenetic modifications of chromatin structure are also required for LTP and memory [9].

Epigenetics have been defined as “*the structural adaptation of chromosomal regions so as to register, signal or perpetuate altered activity states*” [10]. This regulation of chromatin structure enables transcription factors to recognize essential *cis*-regulatory elements and control gene expression. Chromatin remodeling enzymes like the Chromodomain Helicase DNA Binding (CHD) family of ATPases regulate transcription by altering the structure of chromatin [11] and maintaining heritable states of transcription [12]. CHD proteins are well conserved and participate in neural development in both vertebrates [13]–[17] and invertebrates [18].

Kismet (Kis) is the *Drosophila* ortholog of mammalian Chromodomain Helicase DNA Binding Protein 7 (CHD7). Kis was shown to localize to a majority of active transcription sites on salivary gland polytene chromosomes, suggesting that Kis may regulate the transcription of multiple target genes during development [12]. However, the functional relevance of this binding for developmental processes is unclear. Studies in fly larval salivary glands suggest that Kis functions to enable elongation by RNA Polymerase II [12], [19] and functions upstream of the histone H3K4 methyltransferases Trithorax and Ash1 [19], [20]. Kis is required for proper locomotion, memory, axon development, and circadian rhythms [18], [21]. Ubiquitous knockdown of Kis produces flies that are unable to fly and exhibit a prominent postural defect where they hold their wings apart and below their bodies [18]. Collectively, these reports suggest that CHD proteins like Kis may regulate synapse development. Although CHD7 has been implicated in neural crest formation [14] and neurogenesis [15], [22], there are no reports of Kis or CHD7 function in synapse formation or maintenance.

Here we describe the function of Kis in synapse development using the glutamatergic *Drosophila* NMJ. Glutamatergic synapses are highly plastic and exhibit the capacity to alter protein composition and localization to maintain excitability [23]–[26]. Both presynaptic activity [27] and the number and subtype of postsynaptic receptor [28] can be changed to ensure proper synaptic strength. Our results suggest that Kis function is important for motor neuron morphology, synaptic transmission, and the localization of the cell adhesion molecule FasII and postsynaptic proteins including GluRs and Dlg.

## Materials and Methods

### Drosophila Stocks

Fly stocks were maintained at 25°C on standard fly food. The *kis^LM27^* allele was generated by EMS mutagenesis as previously described [29]. *kis^k13416^* and all *Gal4* lines were obtained from Bloomington *Drosophila* Stock Center. *UAS-kis^RNAi.b^* was obtained from Vienna *Drosophila* RNAi Center (VDRC stock #46685). *UAS-Dlg* and *UAS-FasII* were gifts from Vivian Budnik [30], [31]. Kismet-GFP was a gift from Alan Spradling [32].

### Behavioral Testing

25–50 wandering third instar larvae from a non-isogenic background were used for behavioral testing as previously described [33]. Larvae were washed with phosphate solution to rid them of food particles and placed on a non-nutritive agar surface for acclimatization. To measure muscle contractions, an 8.5 cm diameter agar plate was used to provide the larva with a crawling surface. Peristaltic contractions were made using a Leica Mz 125 stereomicroscope for 30 sec per larva. Three trials per larva were performed as previously described [33]–[35].

The crawling behavior of each larva was recorded using Sony DCR-SR47 Handycam with Carl Zeiss optics. The video recording was processed using iMovies software (Apple Inc.) to convert the video file into frames for manual analysis using NIH Image J software. Frame-by-frame analysis of crawling distance was performed manually for each larva. Three trials were performed for each larva. The numerical output generated was used to calculate total distance crawled by the larva.

### Immunohistochemistry

Fly stocks for third instar larval dissections were raised on standard *Drosophila* media. Dissections were performed on Sylgard-coated petri dishes in Roger's Ringer solution (135 mM NaCl, 5 mM KCl, 4 mM MgCl_2_, 1.8 mM CaCl_2_, 5 mM TES, 72 mM sucrose) supplemented with 2 mM glutamate [36]. Animals were fixed for 30 min in either Bouin's fixative (when GluR or Brp antibodies were used) or 4% paraformaldehyde (for all other antibodies). Primary antibodies obtained from the Iowa Developmental Hybridoma Bank included α-Brp/nc82 (1∶50), α-CSP (1∶200), α-DLG (1∶1000), α-FasII (1∶5), α-GluRIIA (1∶100), and α-Syt (1∶100). α-GluRIIB (1∶2000) and α-GluRIIC (1∶5000) antibodies were gifts from Aaron DiAntonio [37]. Mouse monoclonal acetylated tubulin (1∶1000) was obtained from Sigma Aldrich. Rhodamine labeled phallotoxin (1∶200) from Invitrogen was used to label F-actin. Fluorescently conjugated goat α-rabbit or goat α-mouse secondary antibodies (1∶400, Jackson Immunoresearch Labs) were used along with α-HRP (1∶125, Jackson Immunoresearch Labs). Larvae were mounted on slides using Vectashield (Vector Labs). Images from the A3 or A4 6/7 NMJ were obtained using an Olympus Fluoview 1000 laser scanning confocal microscope.

For Kis protein immunolabeling, the *w*; *P{GawB}D42, P{UAS-n-syb-GFP. E}3/TM3, Sb1* (Bloomington stock #9263) was used to highlight motor neurons and α-Kismet (gift from John Tamkun) was used at 1∶100. For Kismet-GFP detection, motor neurons were visualized with *Elav^c155^-Gal4* driving expression of *P{10XUAS-IVS-mCD8::RFP}attP2* (Bloomington Stock #32218) and Kis protein was visualized with Kismet-GFP [32]. Wandering third instar larval brains and body wall muscles were dissected and fixed with paraformaldehyde as described [18]. DAPI was used to visualize nuclei.

GluR cluster sizes were measured as previously described [38] from Z-projected confocal images using NIH ImageJ. Relative fluorescence intensities were quantified by obtaining the mean fluorescence intensity for each NMJ from Z-projected confocal images using Adobe Photoshop (v. CS2) and subtracting the mean fluorescence intensity obtained from an identical area of the muscle or non-NMJ area. Brp density was calculated by counting the number of fluorescent puncta from Z-projected confocal images and dividing by the total NMJ area as indicated by HRP labeling. Apposition of GluRIIC to Brp was measured by quantifying the number of GluRIIC clusters located within 1 µm of a Brp puncta using Z-projected images. Unapposed GluRIIC clusters included any cluster that was located a distance greater than 1 µm from Brp. The unopposed clusters number was divided by the total number of GluRIIC clusters to obtain a percentage.

The number of 6/7 NMJ boutons were counted using the “cell counter” plugin for NIH Image J software. Axonal branches were quantified by manual counting of any bifurcation that included at least two boutons.

### Electrophysiology

Two-electrode voltage clamp recordings were obtained at room temperature from muscle 6 of segments A3 or A4. Third instar larval fillet dissections were performed on Sylgard-coated cover slips in *Drosophila* standard saline (135 mM NaCl, 5 mM KCl, 4 mM MgCl_2_, 1.8 mM CaCl_2_, 5 mM TES, 72 mM sucrose). Muscles were clamped at −60 mV using an Axoclamp 900A amplifier (Molecular Devices). Electrodes filled with 3M KCl that had resistances of 10–20 MΩ were used for intracellular recordings. A 1 Hz stimulus of 10 V was delivered to segmental nerves using an electrode filled with bath saline connected to a Grass S88 stimulator and SIU5 isolation unit (Grass Technologies). Electrophysiological recordings were digitized with a Digidata 1443 digitizer (Molecular Devices) and analyzed using PClamp software (v. 10.4). 180 s of minis per larva were used for analysis. Quantal content was calculated by dividing the eEJC area (nA*ms) by the mEJC area (nA*ms). Intracellular recordings of excitatory junction potentials were also conducted as previously described [39].

### Microarray Studies and data analysis

Microarray analysis was performed by Seqwright (Houston, Tx). 10 total white pre-pupae (as staged by [40]) each from genotypes *Da-Gal4/+* and *Da-Gal4/UAS:kismet RNAi. b* were used. RNA for each sample was isolated by Seqwright from frozen tissues using Qiagen RNeasy Mini kit. The RNA quality of each sample was checked on RNA Nano chip using an Agilent 2100 Bioanalyzer. All RNA samples displayed no degradation with two sharp ribosomal peaks. Affymetrix's GeneChip One-cycle target labeling kit was used for cDNA synthesis and in vitro transcription. Affymetrix GeneChip *Drosophila* Genome 2.0 array was used for this study and the data were processed by Affymetrix Software Expression Console using MAS5.0 Analysis Algorithms to produce. DAT,. CEL, and CHP files of each sample following Seqwright standard procedures.

To produce gene ontology tables, gene lists identified from the microarray analysis were analyzed using the Database for Annotation, Visualization, and Integrated Discovery (DAVID) (http://www.david.abcc.ncifcrf.gov) [41], [42]. Genes whose expression differed significantly (p<0.05) between *Da-Gal4* and *Da-Gal4/UAS:kismet RNAi.b* and showed a twofold or greater change in expression (up- or downregulated) were used for subsequent DAVID analysis.

### Quantitative RT-PCR

For mutant analyses, total RNA was extracted from 8-12 third instar larvae using Trizol as previously described [43], [44]. qRT-PCR was performed in a single step using the iScript One-Step RT-PCR Kit with Sybr Green (Bio-Rad). For RNAi analyses, total RNA was extracted from third instar larval body walls from which the CNS and ventral nerve cord had been removed. After Trizol extraction, the samples were treated with DNase and column purified. Reverse transcription was carried out using the High Capacity cDNA Reverse Transcription kit (Invitrogen). Taqman probes (Applied Biosystems) to *GluRIIA, GluRIIB, GluRIIC* and *Act5c* were used for the real-time reaction using the “StepOnePlus” instrument (Applied Biosystems). Appropriate controls were maintained throughout this study. 100 ng of RNA was added to each reaction. For mutant analysis, ΔC(t) values were calculated by subtracting the C(t) value of the GluR-specific reaction from the C(t) value obtained for GAPDH. For RNAi analysis, *Act5c* was used as the reference gene and analysis was performed by calculating fold change in expression (RQ) from ΔΔC(t) values. Each reaction was performed in triplicate and three biological replicates were performed and used for data analysis.

### Statistical Analyses

Statistics were performed using Graph Pad Prism (v 5.01). Statistical comparisons included student's t-tests for analysis of *kismet* mutant phenotypes, and, for RNAi analysis, a one-way ANOVA with post-hoc Tukey or Games-Howell tests depending on the variation between data sets. Statistical significance in figures is represented by:* = p<0.05, ** = p<0.001, and *** = p<0.0001. Error bars represent the standard error of the mean (SEM). Summary statistics including means, SEM, number of animals, and additional information can be found in Table S1.

## Results

### Kismet is localized to motor neuron and muscle nuclei

**Figure 1:**
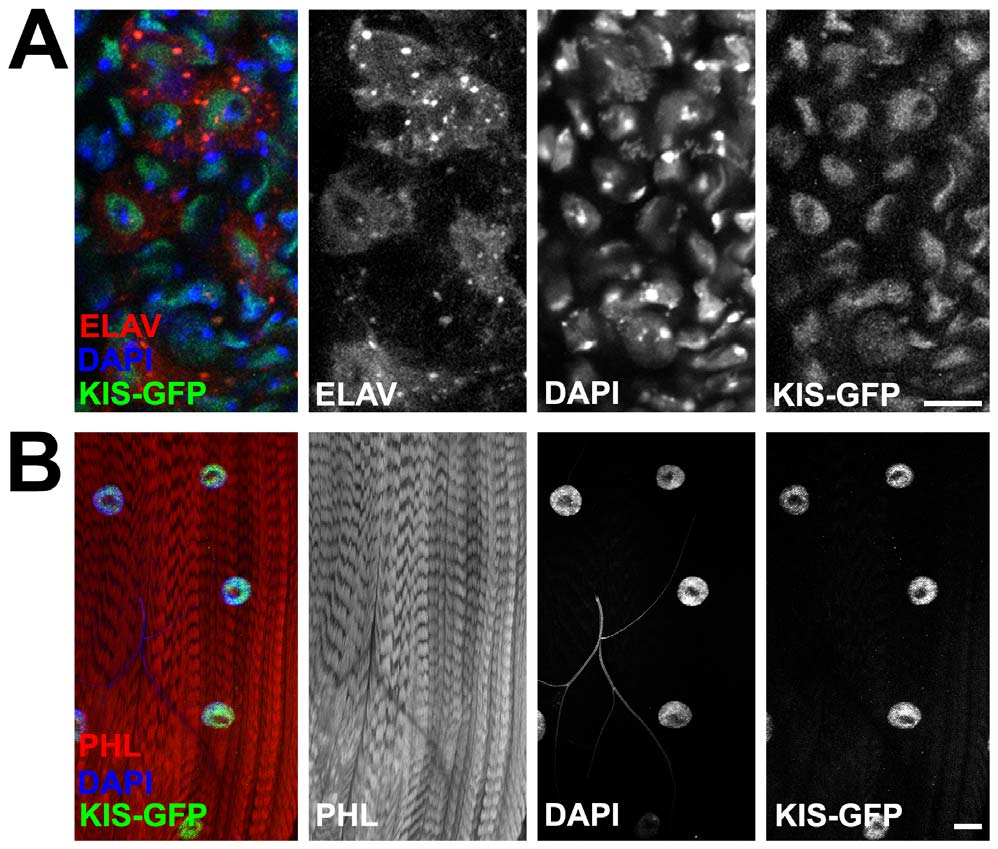
Kis localizes to the nucleus of motor neurons and muscles. (**A**) Confocal images of Kis-GFP expression in midline of third instar larval ventral nerve cord expressing RFP in all neurons using the *Elav^c155^-Gal4* driver. Neurons are labeled in red (Elav), nuclei in blue (DAPI), and Kis in green (Kis-GFP). Note presence of Kis-GFP in neuron nuclei. Right panels show individual channels. Scale bar  = 10 µm. (**B**) Confocal images of Kis-GFP expression in multi-nucleated muscle cells of muscles 6 and 7 in third instar larval NMJs. Muscles are labeled with phalloidin (PHL, red), muscle nuclei in blue (DAPI), and Kis in green (Kis-GFP). Note presence of Kis-GFP in muscle nuclei. Right panels show individual channels. Scale bar  = 50 µm.

We sought to further explore the role Kis may play in synaptic development using the *Drosophila* NMJ as a model system. Kis has been shown to regulate RNA polymerase II-mediated transcription [12], [19]. Therefore, we first examined the localization of Kis protein in third instar larvae using both immunolabeling for the long isoform of Kis (Kis-L; [12]) and a Kis-GFP protein trap stock [32]. We had previously shown that Kis is localized in the nuclei of multiple cells in the ventral nerve cord (VNC) of third instar larvae [18]. To determine first that Kis localizes to neurons, we labeled neurons in the third instar VNC by driving expression of a membrane-tagged RFP with *elav-Gal4* and focusing on the midline of the VNC. We then analyzed the localization of Kis-GFP within this tissue. As previously reported [18], Kis localized to multiple nuclei within the VNC including the nuclei of multiple neurons (Figure 1A). To verify that these neurons were motor neurons, we labeled motor neurons by driving *n-syb-GFP* with the motor neuron-specific *D42-Gal4* driver. We observed that Kis localized to the nuclei of VNC motor neurons (Figure S1A). Using both methods, we also observed Kis localized in multinucleated postsynaptic muscles (Figure 1B and Figure S1B). We did not observe Kis localized outside of the nucleus in either analyses. These results are consistent with the role of Kis as a transcription regulator [19] and indicate that Kis may regulate both pre- and postsynaptic components of the NMJ via its nuclear actions.

### Loss of Kismet function slightly alters synaptic morphology

**Figure 2:**
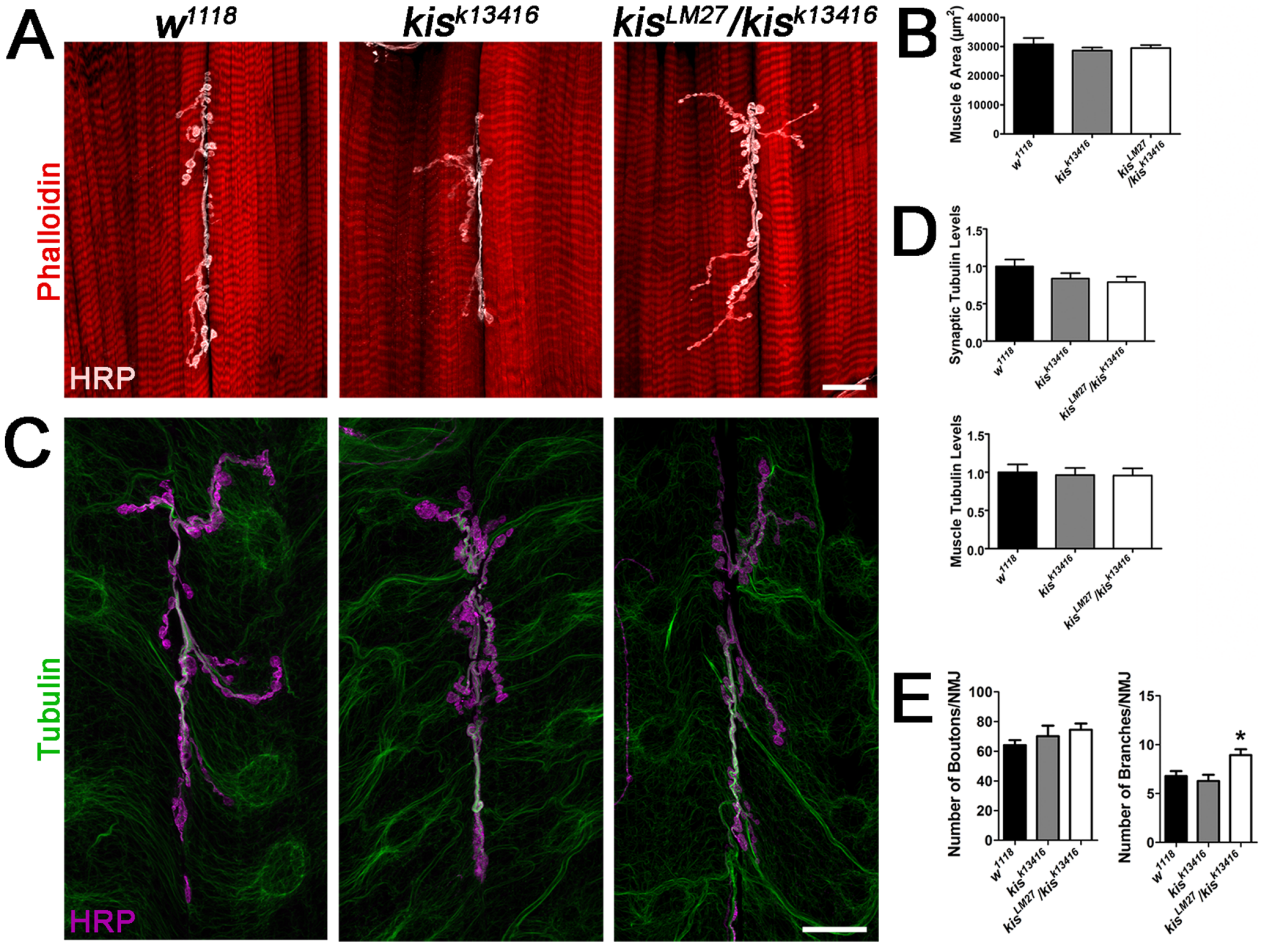
Kis negatively affects growth of the presynaptic motor neuron but does not alter cytoskeletal proteins at the NMJ. (**A**) Confocal images of third instar larval NMJs, muscles 6 and 7, labeled with α-HRP (white) to detect presynaptic neuronal membranes and phalloidin (red). Scale bar  = 20 µm (**B**) Quantification of muscle 6 sizes in *w^1118^*, *kis^k13416^*, and *kis^LM27^/kis^k13416^*. (**C**) Confocal micrographs of 6/7 NMJs from third instar larvae immunolabeled with α-HRP (magenta) and α-acetylated tubulin (green). Scale bar  = 20 µm (**D**) Quantification of mean relative synaptic acetylated tubulin levels (top histogram) and mean relative muscle acetylated tubulin levels (bottom histogram). (**E**) Quantification of total boutons (left histogram) and branches (right histogram) per 6/7 NMJ.

Both the actin and microtubule cytoskeletons are important for the structure, stability, and the maintenance of synapses (for review see [45]). The microtubule cytoskeleton is essential for both synaptic function and muscle morphology as it forms and provides stability to structural components of neurons and synapses [46], [47]. To determine whether *kis* influences the dynamics of the synaptic cytoskeleton like other chromatin remodeling enzymes [48]–[50], we labeled both the actin and microtubule cytoskeletons at the NMJ using two *kis* mutant alleles. *kis^k13416^* is a weak adult-viable hypomorph due to the insertion of a transposable element in the 5′ end of the gene [51] while *kis^LM27^* is a strong protein null allele [29]. Because *kis^LM27^* nulls are early larval lethal, we used the heteroallelic *kis^LM27^/kis^k13416^* combination, which is pupal lethal. Mutations in *kis* do not affect gross muscle morphology as indicated by phalloidin, which labels F-actin [52], or the size of ventral body wall muscles 6 (Figures 2A–B) and 7 (data not shown, *w^1118^* = 17930±1173 µm^2^, n = 9; *kis^k13416^* = 16980±543.5 µm^2^, n = 9, p = 0.47; *kis^LM27^/kis^k13416^* = 17150±682.8 µm^2^, n = 8, p = 0.61). Similarly, there were no significant differences in the levels of acetylated tubulin at the synapse or in the muscle of *kis* mutants compared with controls (Figures 2C–D). Post-translational acetylation of α-tubulin occurs at stable microtubules [53].

Given the role of transcriptional regulators such as AP-1 [54] and Zfh1 [55] in NMJ development and morphology, we next examined NMJ morphology in *kis* mutants and in animals after ubiquitous knockdown of *kis* using the *UAS/Gal4* system [56]. Ubiquitous knockdown of Kis was achieved using the *da-Gal4* driver to express a *UAS:kismet RNAi.b* transgene (*da>kis^RNAi.b^*), which was previously shown to strongly reduce Kis protein levels (roughly 90% overall) and severely reduce adult motor function [18]. While we noticed a trend indicating the numbers of boutons per 6/7 NMJ were slightly increased in both *kis* mutants compared with controls (Figure 2E), this increase was not statistically significant. However, ubiquitous knockdown of *kis* resulted in a significant increase in the total number of boutons in *da>kis^RNAi.b^* (data not shown, 94.4545±5.9043, p<0.001) when compared to outcrossed controls *da-Gal4/+* (57.3636±4.3469) and *UAS-kis^RNAi.b^/+* (70.7273±4.8450, p<0.001). We also observed a significant increase in the total number of branches in *kis^LM27^/kis^k13416^* larvae, but not *kis^k13416^* larvae compared to *w^1118^* controls (Figure 2E). Taken together, these data suggest that *kis* influences NMJ morphology but does not affect ventral body wall muscle size or muscle morphology.

### Kismet selectively regulates the levels of the cell adhesion molecule FasII and the postsynaptic protein Dlg

Several synaptic proteins serve as markers of synaptic plasticity at the *Drosophila* NMJ such as Bruchpilot (Brp), Cysteine String Protein (CSP), Discs Large (Dlg), Fasciclin II (FasII), and Synaptotagmin (Syt). To determine if the loss of *kis* affects other synaptic proteins, we first examined the number of active zones, sites of neurotransmitter release, by immunolabeling for the T-bar associated protein Brp [57], [58]. We did not observe any significant difference in the density of Brp puncta in either *kis* mutant compared to control larvae (Figure S2A). Similarly, mutations in *kis* did not produce a significant change in relative fluorescence for the synaptic vesicle markers CSP and Syt (Figure S2B–C).

Interestingly, we observed a significant increase in synaptic levels of both FasII and Dlg in *kis* mutants (Figure S3). Dlg is a postsynaptic scaffolding protein that stabilizes GluRIIB-containing clusters [59] while FasII is an activity-dependent cell adhesion molecule [60]. *kis* mutant larvae exhibited a significant increase in the relative fluorescence intensity of Dlg compared to *w^1118^* controls (Figure S3A). Similarly, FasII immunoreactivity was significantly increased in *kis* mutants (Figure S3B) compared to *w^1118^* controls. Taken together, these data suggest that Kis may serve as a negative regulator of FasII and Dlg at the synapse but does not significantly affect multiple presynaptic proteins including the functional presynaptic markers Brp, CSP, and Syt.

### Kismet positively regulates postsynaptic GluR localization in larval muscles

Recently our labs independently identified *kis* as necessary for GluR localization/expression via two separate approaches. First, through a microarray analysis of *kis* loss of function pupae (Tables S2–S3), we found that the expression level of the GluR subunits *GluRIIB* and *GluRIIC* were significantly reduced. Second, we also identified mutations in *kis* in a forward genetic screen for phenotypes that affect the localization of GluRIIA in third instar larval muscles (data not shown). To further explore the connection between *kis* function and *GluR* expression, we next examined ionotropic GluRs (iGluRs) at third instar larval NMJ in *kis* loss of function mutants and after ubiquitous knockdown of Kis. There are five iGluR subunits expressed in *Drosophila* muscles including GluRIIA, GluRIIB, GluRIIC, GluRIID, and GluRIIE. These subunits are most similar in structure and function to vertebrate AMPA and kainate receptors [37], [61], [62]. The five subunits form two distinct tetrameric GluR complexes, which contain either GluRIIA or GluRIIB, plus the remaining essential GluRIIC, -IID, and -IIE subunits [37], [62].

**Figure 3:**
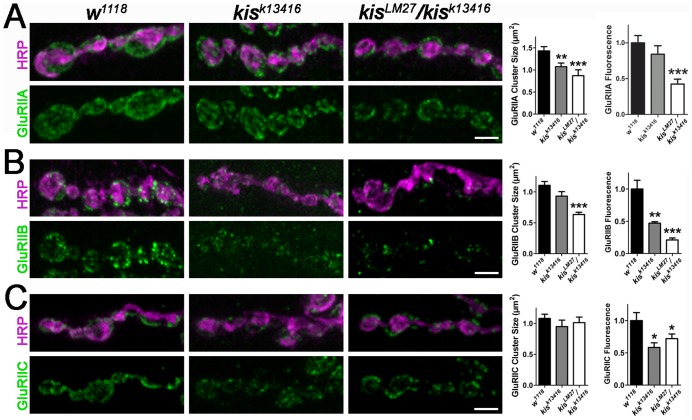
Kis positively regulates localization of postsynaptic glutamate receptors. High resolution confocal images of 6/7 NMJs from third instar larvae immunolabeled with α-HRP (magenta) and α-GluRIIA (**A**, green), α-GluRIIB (**B**, green), or α-GluRIIC (**C**, green). Scale bar  = 5 µm. Histograms show quantification of GluR cluster sizes (left histograms) and mean relative GluR fluorescence (right histograms) in genotypes listed.

Both *kis* loss of function mutants exhibited a significant decrease in GluRIIA cluster size compared to *w^1118^* controls (Figure 3A). GluR cluster size has been shown to correlate with receptor function [38] and is used as an indicator of the number of postsynaptic receptors [59]. This corresponded to a 57.6% reduction in mean relative fluorescence intensity in *kis^LM27^/kis^k13416^* mutants compared to controls. Further, there was a small but non-significant reduction in GluRIIB cluster size in *kis^k13416^* homozygous larvae and a significant reduction in GluRIIB cluster size in *kis^LM27^/kis^k13416^* mutant larvae compared to *w^1118^* controls (Figure 3B). Although there was no significant change in GluRIIC cluster size in either *kis* mutants (Figure 3C), there was a significant reduction in mean relative GluRIIC fluorescence levels in both *kis* mutants compared to *w^1118^* controls (Figure 3C). The significant reduction in GluRIIC relative fluorescence without a corresponding reduction in GluRIIC cluster size indicates there is likely a loss of GluRIIC puncta in *kis* mutants. Similar to our mutant analyses, knockdown of *kis* in all tissues using the *da-Gal4* driver also resulted in significant decreases in GluRIIA, GluRIIB, and GluRIIC cluster sizes (Figure S4). Collectively, these data suggest that Kis positively influences synaptic levels of iGluRs.

Given the role of Kis in transcription elongation [12], [19], we next analyzed the relative levels of *iGluR* mRNAs in both *kis* mutants and RNAi knockdowns. We did not observe a significant decrease in *GluRIIA, GluRIIB*, or *GluRIIC* transcript levels in *kis^k13416^* homozygous mutant larvae, *kis^LM27^/kis^k13416^* larvae, or ubiquitous Kis knockdowns when compared to controls (data not shown). Taken together, these data suggest that Kis may be involved in the localization and clustering of postsynaptic iGluR subunits by influencing other synaptic transcripts but may not be involved in the transcriptional regulation of *iGluR* subunits. Thus, mutations in *kis* may secondarily affect iGluRs as a consequence of other synaptic perturbations.

Because we observed significant effects on both Dlg and FasII expression levels in *kis* mutants (Figure S3), we sought to determine whether the increased synaptic levels of Dlg or FasII could explain the loss of postsynaptic iGluRs observed in *kis* mutants. We overexpressed *Dlg* or *FasII* in all tissues using the *actin5c-Gal4* driver. Ubiquitous overexpression of *Dlg* or *FasII*, however, did not significantly affect GluRIIA cluster sizes (Figure S5) suggesting that the relative increase in either Dlg or FasII likely did not contribute to the loss of iGluRs observed in *kis* mutants.

### Kismet positively regulates neurotransmission and quantal content

**Figure 4:**
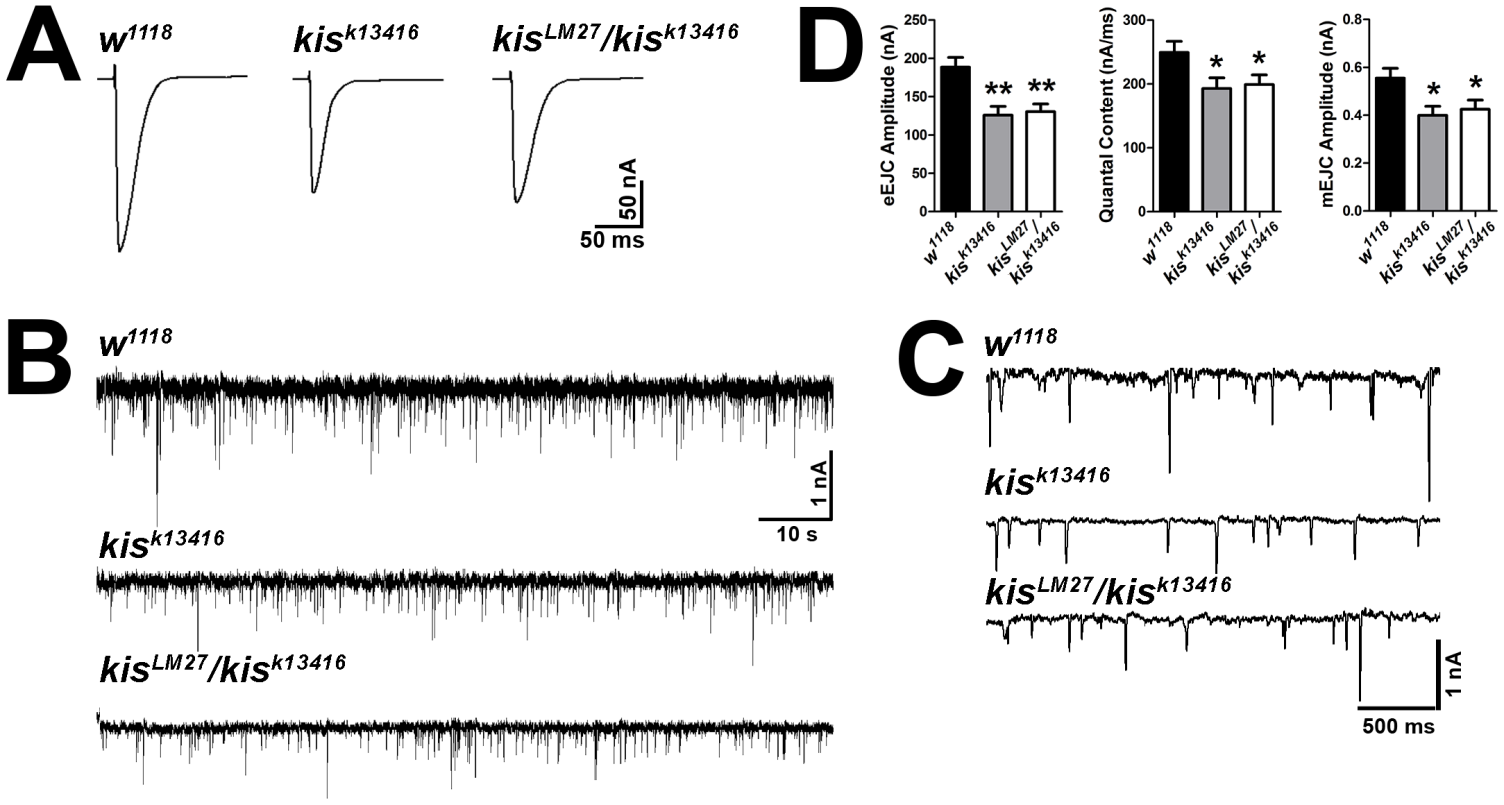
Kis positively regulates evoked synaptic transmission. (**A**) Representative evoked excitatory junctional currents (eEJCs) from control and *kis* mutants. Muscles were voltage clamped at −60 mV and a 10V, 1 Hz stimulus was applied to the presynaptic motor neuron. (**B–C**) Representative mEJC traces from control and *kis* mutants. (**D**) Quantification of eEJC amplitudes, quantal content, and mEJC amplitudes.

The reduction in synaptic iGluRs, increase in synaptic levels of Dlg and FasII, and change in morphology of the presynaptic motor neuron in *kis* mutants collectively suggest that Kis may regulate synaptic development without significantly affecting i*GluR* mRNA levels. We therefore examined neurotransmission using two-electrode voltage clamp and found significant reductions in both evoked excitatory junctional current (eEJC) amplitudes, miniature excitatory junctional current (mEJC) amplitudes, an indicator of spontaneous neurotransmission, and quantal content in *kis* mutants compared with controls (Figure 4). There were no significant differences in mEJC frequency (data not shown, *w^1118^* = 3.438±0.373 Hz, n = 13, *kis^k13416^* = 3.423±0.827 Hz, n = 12, p = 0.986, *kis^LM27^/kis^k13416^* = 3.014±0.450 Hz, n = 12, p = 0.473). Similarly, knockdown of Kis in all tissues resulted in a significant reduction in the amplitude of excitatory junction potentials (EJPs, data not shown). These data suggest that *kis* function is important for neurotransmission at the *Drosophila* larval NMJ.

### Kismet affects the alignment of postsynaptic receptors with presynaptic active zones

**Figure 5:**
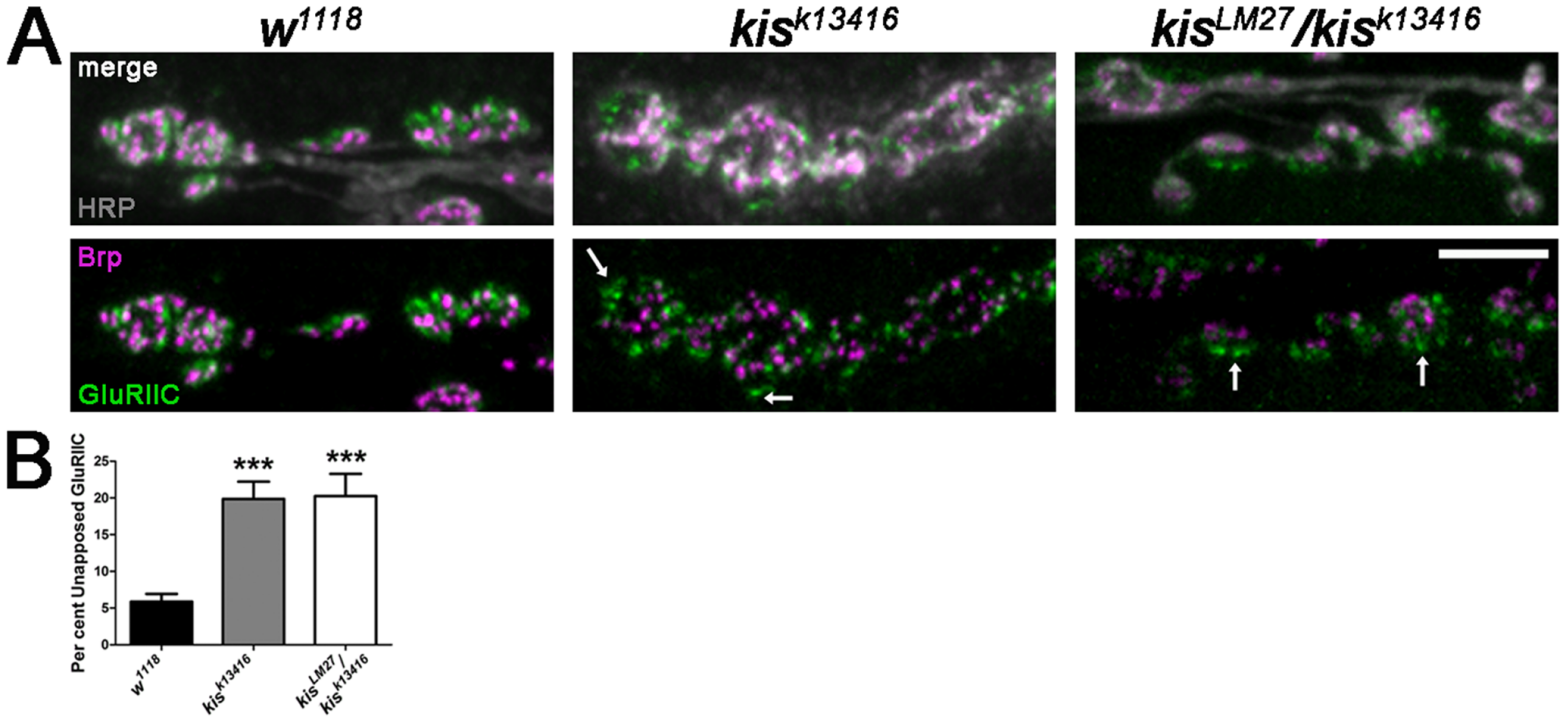
Kis influences the apposition of postsynaptic GluRs with presynaptic active zones. (**A**) High resolution confocal images of third instar larval NMJs, muscles 6 and 7, immunolabeled with α-HRP (gray), α-Brp (magenta), and α-GluRIIC (green). Arrows indicate examples of GluRIIC clusters that are unapposed to an active zone as indicted by Brp immunlabeling. Scale bar  = 5 µm. (**B**) Quantification of the per cent of GluRIIC clusters that are unopposed to a Brp puncta.

Proper synaptic function requires the precise localization of both pre- and postsynaptic components such that each postsynaptic iGluR cluster is directly apposed to a presynaptic active zone [63]. Because of the defects we observed in iGluR clustering (Figure 3C) and synaptic transmission, we quantified the number of iGluR clusters (visualized by GluRIIC) that were unopposed to active zones as indicated by Brp puncta. Mutations in *kis* do not affect the density of Brp puncta (Figure S2A) but may affect the number of postsynaptic GluRIIC clusters (Figure 3C). There was a significant increase in the number of GluRIIC clusters unapposed to Brp puncta in *kis* mutant larvae compared to control larvae (Figure 5) suggesting that *kis* mutants contain fewer functional synapses. Collectively, these data indicate that Kis, by affecting transcription of synaptic target genes, may function to regulate the relative distribution and localization of specific synaptic proteins to facilitate proper synaptic development and function.

### Kismet exhibits tissue-specific effects on iGluR clustering and locomotion

**Figure 6:**
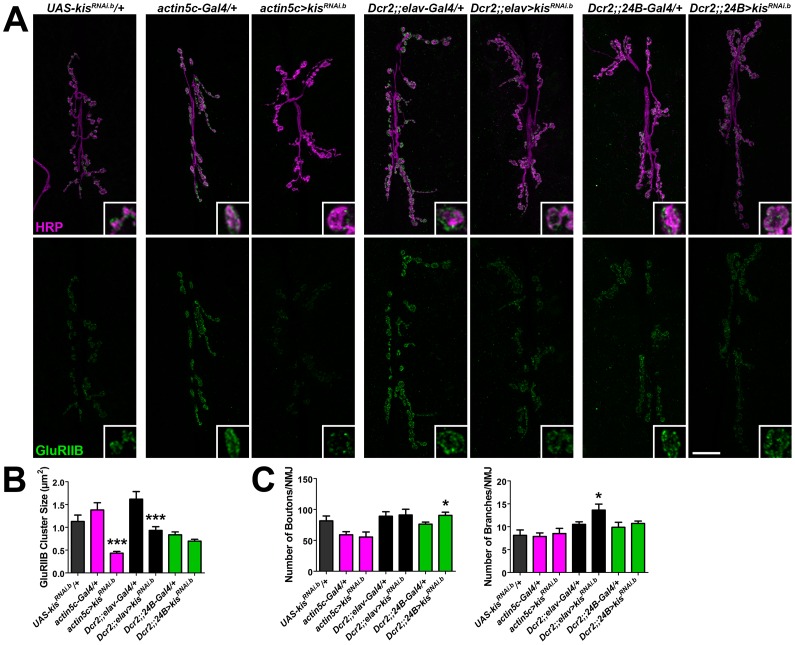
Kis is important pre- and postsynaptically for NMJ development. (**A**) Confocal micrographs of third instar larval 6/7 NMJs immunolabled with α-HRP (magenta) and α-GluRIIB (green). Insets show high magnification image of a single terminal bouton. Scale bar  = 20 µm. (**B**) Quantification of GluRIIB cluster size in µm^2^ in genotypes listed indicates that knockdown of *kis* in all cells or presynaptic neurons but not postsynaptic muscles results in a significant reduction in GluRIIB cluster size. (**C**) Quantification of the number of 6/7 NMJ boutons (left) and branches (right) in the genotypes listed.

Kis is strongly expressed in both presynaptic motor neuron nuclei as well as in muscle nuclei (Figure 1). To determine the tissue-specific effects of *kis* loss of function on NMJ development, we used two drivers including *Dcr2;;elav-Gal4* to knockdown Kis in neurons and *Dcr2;;24B-Gal4* to knockdown Kis in postsynaptic muscles. To validate the effects we observed with Kis knockdown using *da-Gal4*, we utilized a second *Gal4* line (*actin5c-Gal4*) as a positive control for ubiquitous knockdown. We focused our analysis on GluRIIB since it exhibited the most severe reduction at *kis^LM27^/kis^k13416^* mutant NMJs (Figure 3B). Consistent with our previous results using *da-Gal4*, ubiquitous Kis knockdown with *actin5c-Gal4* resulted in a significant reduction in GluRIIB cluster size (Figure 6B). Knockdown of Kis in neurons but not muscles produced both a significant decrease in GluRIIB cluster size (Figure 6B) and an increase in motor neuron branches (Figure 6C). In contrast, knockdown of Kis in muscles alone resulted in a significant increase in synaptic boutons (Figure 6C). Taken together, our data suggests that Kis may regulate synaptic gene products that function in the motor neuron to help properly localize postsynaptic iGluRs. Kis gene products function in both motor neurons and muscle to regulate NMJ morphology.

**Figure 7:**
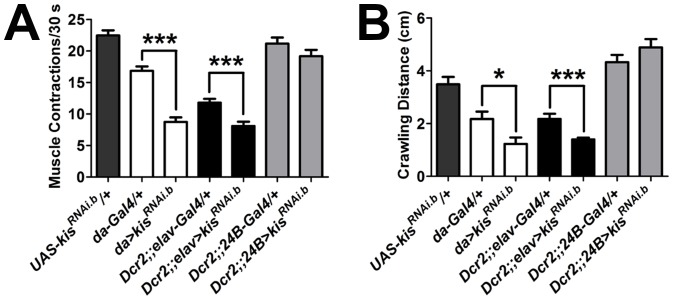
Kis functions predominantly in motor neurons to influence larval behavior. (**A**) Quantification of the number of muscle contractions per 30 seconds from third instar larvae in genotypes listed. Muscle contractions were quantified as the number of full body, entire peristaltic waves (originating forward or backward) generated by each third instar larvae. The mean muscle contraction was determined from three consecutive trials per larvae performed for genotypes listed. (**B**) Quantification of crawling distance in cm/min from third instar larvae in genotypes listed. Manual analysis of 60 video frames per larvae. Three trials per larvae were carried out to calculate the average. The mean was used as a single data point in the analysis.

To extend our findings into functional behavior and to further analyze the tissue-specific effects of Kis, we next analyzed the effects of ubiquitous and tissue-specific knockdown of Kis on larval locomotive behavior by examining the frequency of muscle contractions and total crawling distance. Larval locomotion is the result of central pattern generators producing bursts of neuronal activity [64], which lead to contractions of both dorsal and ventral body wall muscles [65]. Thus, larval locomotion relies, at least in part, on proper glutamatergic neurotransmission [66]. There was a significant reduction in both the frequency of muscle contractions (Figure 7A) and distance crawled (Figure 7B) by larvae after ubiquitous knockdown of Kis compared to controls. Presynaptic knockdown of Kis, but not muscle-specific knockdown of Kis, also led to a significant reduction in the frequency of muscle contractions produced and total distance crawled by these larvae compared to controls (Figure 7). Taken together, these data suggest that Kis influences both the function of and development of the glutamatergic NMJ.

## Discussion

Our results are the first to demonstrate a role for Kis in synaptic development and function of the *Drosophila* NMJ. The functional role is likely related to Kis influencing transcription of target genes involved in iGluR localization and apposition to presynaptic release sites. *Kis* mRNA is expressed ubiquitiously early in embryogenesis [67] and Kis protein has been detected in larval salivary glands [12], central nervous system [18], retina [29], and in the adult wing [68]. Our results indicate that Kis is localized to ventral motor neuron and muscle nuclei (Figure 1, Figure S1). Although we did not find that Kis directly regulated iGluR transcript levels, the proposed role of Kis in transcription elongation [19] implies that Kis-mediated transcriptional regulation of target genes relevant to synaptic function could collectively regulate synapse development. Indeed, our microarray results from *kis* loss of function pupae identified many potential target genes that encode synaptic proteins and transcription factors that we have not tested in this particular study (Tables S2–S5).

### Kismet regulates synapse function and morphology

Kis contains an ATPase domain, two chromodomains, and a BRK domain [67], [69] suggesting that it promotes chromatin remodeling. Chromatin remodeling enzymes including those that regulate DNA methylation, histone modifications, and nucleosome positioning may positively or negatively influence transcription in response to developmental cues and neural activity [70]. In mammals, chromatin modifying enzymes including nBAF complexes containing BAF53b [71] and histone deacetylase 2 (HDAC2) [72] regulate hippocampal dendritic density and synaptic activity. We find that reduced *kis* levels result in an overgrowth of the presynaptic motor neuron (Figure 2) and a significant reduction in the amplitudes of both evoked and spontaneous (i.e. mEJCs) synaptic currents and quantal content (Figure 4). The reduction in mini amplitudes may reflect the reduction in synaptic iGluRs (Figure 3) we observe in *kis* mutants. The endocytosis mutants *brain tumor* [73] and *dap160* [74], [75] exhibit increases in the number of synaptic boutons and decreases in evoked EJPs. Although mEJP amplitudes are significantly increased in these mutants [73], [75], mutations that affect endocytosis differentially influence spontaneous activity. The G-actin sequestering protein, twinfilin, positively regulates endocytosis and localization of GluRIIA but does not influence spontaneous neurotransmission [76]. Our collective data suggest that mutations in *kis* lead to a defect at the level of vesicular release, vesicular loading, or synaptic vesicle endocytosis, which over time may lead to reduced receptor clustering, larval motor behavior, and other synaptic changes.

### Kismet regulates the localization of synaptic proteins including Dlg, FasII, and iGluRs

Synaptic transmission is also affected by mutations that disrupt the apposition of GluRs to Brp. *Microtubule star* (mts), which encodes the catalytic subunit of protein phosphatase 2A [77], and *rab3* mutants [78] both exhibit a significant increase in the percentage GluRIIC clusters unopposed to Brp similar to *kis* mutants (Figure 5). *mts* positively regulates evoked neurotransmission and mEJP amplitudes [77] similar to Kis. Conversely, *rab3* is important for short-term facilitation but does not influence mEJP amplitudes or quantal content [78]. These data indicate that iGluR apposition to Brp is affected by disparate synaptic mechanisms and may be a general indicator of altered synaptic signaling. Since we observe a significant decrease in GluRIIC fluorescence without a significant decrease in GluRIIC cluster size in the *kis* mutants we analyzed, we hypothesize that 1) there are fewer GluRIIC clusters (decreased fluorescence) and 2) the GluRIIC clusters that are present are not properly aligned with presynaptic active zones.

Changes in synaptic function may lead to compensatory changes in the synapse over time. We observe an increase in the relative fluorescence of Dlg and FasII in *kis* mutants (Figure S3). An increase in the levels of FasII over time would promote the formation of additional synapses through an increase in the total number of boutons [30], which is what we observe in *kis* mutants. While loss of *kis* in third instar larvae results in a reduction in iGluR cluster sizes (Figure 3, Figure S4), we did not observe a significant effect on *GluRIIA*, *GluRIIB*, and *GluRIIC* transcripts in *kis* mutants or as a result of ubiquitous Kis knockdown. Although this result appears contradictory to our microarray results (Table S3), *iGluR* transcript levels were measured in third instar mutant larvae while Kis knockdown pupae were used for the microarray. Given that Kis is involved in transcription elongation and epigenetic control of transcription [12], [19], our data suggests that the loss of GluRs from the synapse may be secondary to other synaptic changes instead of a decrease in Kis-mediated *iGluR* transcription. Indeed, our tissue-specific analyses suggest that the decreased iGluR levels in postsynaptic muscles may be due to defective signaling from presynaptic motor neurons (Figure 6).

### Synaptic mechanisms underlying CHARGE Syndrome

*kis* is the *Drosophila* homolog of *CHD7* in humans [18]. Haploinsufficiency of *CHD7* is associated with CHARGE syndrome, an autosomal dominant disorder characterized by multiple developmental symptoms that include hypotonia, limb abnormalities, and cranial nerve defects [79]–[83]. Individuals with CHARGE syndrome can display delayed or impaired motor coordination and adaptive motor skills, as well as musculoskeletal anomalies [79], [84].

Mechanisms underlying the symptoms associated with CHARGE syndrome are currently not clear. We have previously shown that *k*is loss of function leads to defective adult motor behavior. Ubiquitous knockdown of *kis* produces flies that are unable to fly and exhibit a prominent postural defect where they hold their wings apart and below their bodies [18]. This postural defect is reminiscent of defects associated with muscles and neuromuscular synaptic transmission and is consistent with hypotonia related postural problems often observed in CHARGE individuals [84]. Our current data suggest that defective synaptic function translates to defective motor behavior in animals with decreased *kis* function (Figure 7). Loss of *kis* function leads to defects in motor coordination as assayed through larval crawling. The aberrations observed in synaptic transmission and synaptic development in *kis* mutant larvae may also be present in mammals with decreased *CHD7* function, including CHARGE individuals.

In summary, we describe, for the first time, the function of the CHD7 homolog, Kis, in synaptic development. Our results suggest that Kis function is important for proper synaptic transmission, localization of synaptic proteins, motor neuron morphology, and larval motor behavior. We suggest that the aberrant synaptic activity in *kis* mutants leads to decreased levels of and altered localization of postsynaptic GluRs over time, further affecting motor behavior and NMJ function.

## Supporting Information

Figure S1 **Kis localizes to the nucleus of motor neurons and muscles.** (**A**) Confocal images of third instar larval ventral nerve cord immunolabeled with α-Kis-L (red), DAPI (blue), and *UAS-n-syb-GFP* driven by the motor neuron specific driver *D42-Gal4* (green). Neurons are labeled in green (Elav), nuclei in blue (DAPI), and Kis-L in red. Note presence of Kis-L in motor neuron nuclei. Right panels show individual channels. Scale bars  = 20 µm. (**B**) Confocal images of third instar larval NMJs, muscles 6 and 7, immunolabeled with α-Kis-L (red), α-Phalloidin (green) and DAPI (blue). Right panels show individual channels. Scale bar  = 100 µm.
(TIF)

Figure S2 **Kis does not alter Brp, CSP, or Syt levels.** High resolution confocal images of 6/7 third instar larval NMJs immunolabled with α-HRP (magenta) and α-Brp (**A**, green), α-CSP (**B**, green), or α-Syt (**C**, green). Top panels show merged images. Quantification of relative fluorescence is shown in the right subpanels. Scale bar  = 5 µm.
(TIF)

Figure S3 **Kis negatively influences synaptic Dlg and FasII levels.** (**A**) High resolution confocal micrographs 6/7 NMJs from third instar larvae immunolabeled with α-HRP (magenta) and α-Dlg (green). Right histogram shows quantification of mean relative Dlg levels in genotypes listed. (**B**) Confocal images of third instar larval NMJs, muscles 6 and 7, immunolabeled with α-HRP (magenta) and α-FasII (green). Right histogram shows quantification of mean relative Dlg fluorescence in genotypes listed. Scale bar  = 5 µm.
(TIF)

Figure S4 **Ubiquitous knockdown of Kis regulates postsynaptic glutamate receptor localization.** (**A**) High resolution confocal images of third instar larval muscles 6 and 7 NMJs immunolabeled with α-HRP (magenta) and α-GluRIIA (green). Quantification of GluRIIA cluster size in µm^2^ shown in right histogram. (**B**) Confocal micrographs of third instar larval muscles 6 and 7 NMJs immunolabeled with α-HRP (magenta) and α-GluRIIB (green). Right histogram shows quantification of GluRIIB cluster size in µm^2^. (**C**) Confocal images of third instar larval muscles 6 and 7 NMJs immunolabeled with α-HRP (magenta) and α-GluRIIC (green). Quantification of GluRIIC cluster size in µm^2^ shown in right histogram. Scale bar  = 5 µm.
(TIF)

Figure S5 **Ubiquitous overexpression of Dlg and FasII does not alter GluR localization.** (**A**) Confocal images of third instar larval 6/7 NMJs immunolabeled with α-HRP (magenta) to label presynaptic motor neurons and α-GluRIIA (green). *Dlg* and *FasII* were overexpressed ubiquitously using the *Actin5c-Gal4* driver. Insets show high magnification image of a single terminal bouton. Scale bar  = 20 µm. (**B**) Quantification of GluRIIA cluster size in µm^2^. (**C**) Quantification of the number of 6/7 NMJ boutons (left) and branches (right) in the genotypes listed.
(TIF)

Table S1 **Summary statistics for all data.**
(XLSX)

Table S2 **Selected Gene Ontology clusters related to nervous system function mis-regulated in response to** ***kismet*** **knockdown.**
(DOCX)

Table S3 **Synapse target genes showing decreased expression.**
(DOCX)

Table S4 **Neurotransmitter receptor genes showing decreased expression.**
(DOCX)

Table S5 **Neurotransmitter receptor genes showing increased expression.**
(DOCX)
