# Supplementary figures and images for: Kismet Positively Regulates Glutamate Receptor Localization and Synaptic Transmission at the Drosophila Neuromuscular Junction
Source: PLoS One. 2014 Nov 20;9(11):e113494. doi: 10.1371/journal.pone.0113494 (PMC4239079; doi:10.1371/journal.pone.0113494)

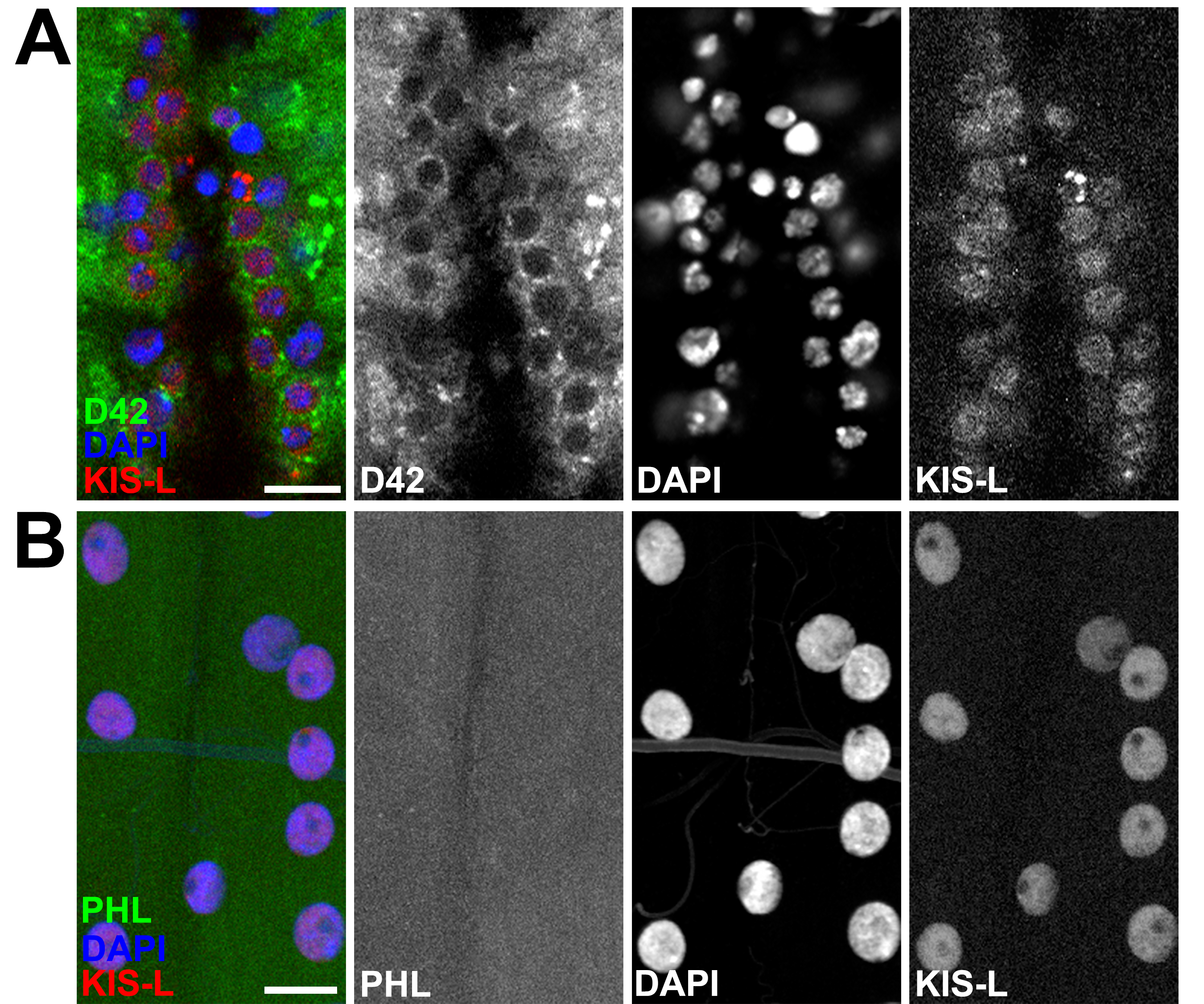

Supplement: Figure S1 — Kis localizes to the nucleus of motor neurons and muscles. (A) Confocal images of third instar larval ventral nerve cord immunolabeled with α-Kis-L (red), DAPI (blue), and UAS-n-syb-GFP driven by the motor neuron specific driver D42-Gal4 (green). Neurons are labeled in green (Elav), nuclei in blue (DAPI), and Kis-L in red. Note presence of Kis-L in motor neuron nuclei. Right panels show individual channels. Scale bars = 20 µm. (B) Confocal images of third instar larval NMJs, muscles 6 and 7, immunolabeled with α-Kis-L (red), α-Phalloidin (green) and DAPI (blue). Right panels show individual channels. Scale bar = 100 µm. (TIF) [file pone.0113494.s001.tif]

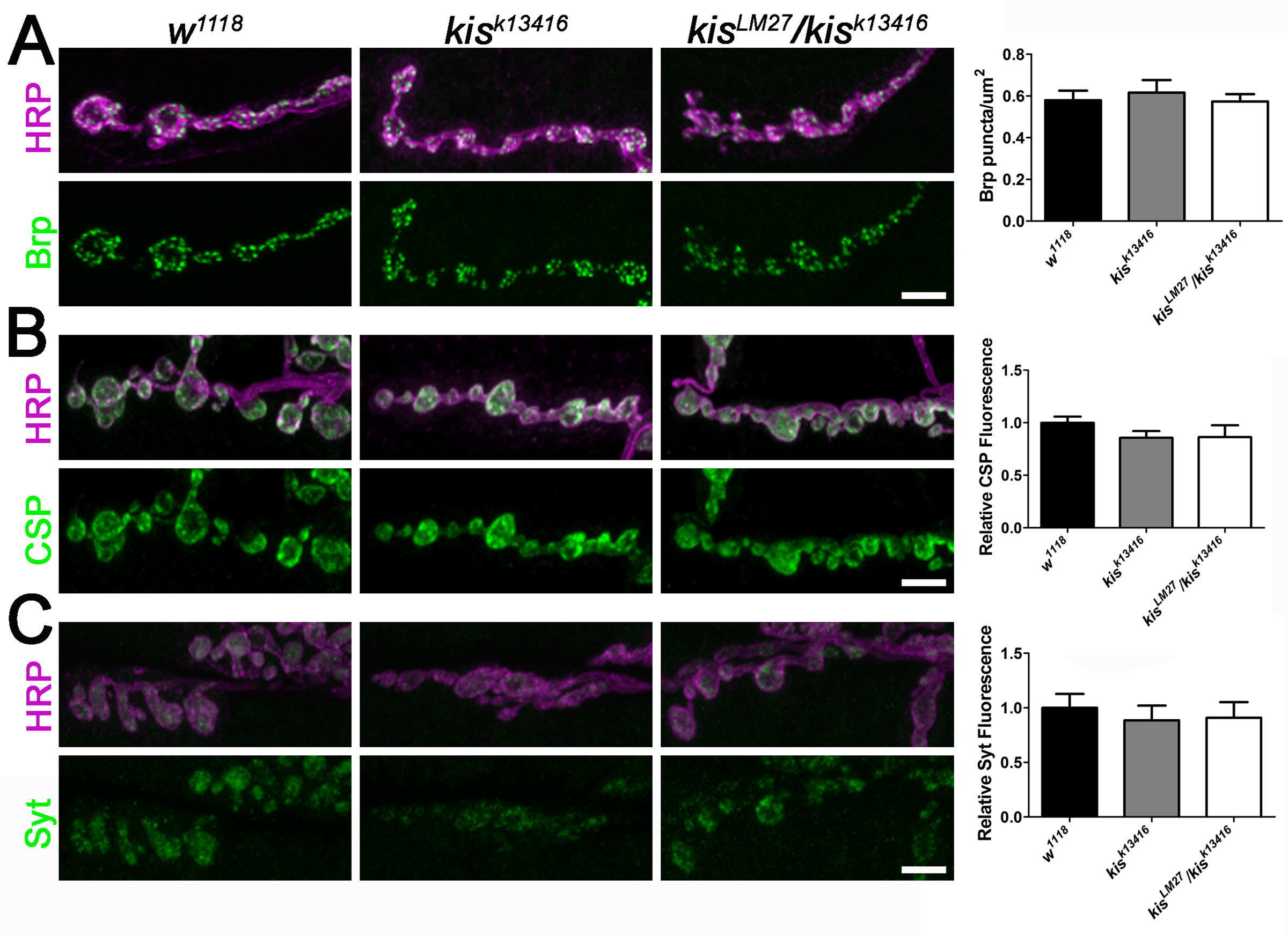

Supplement: Figure S2 — Kis does not alter Brp, CSP, or Syt levels. High resolution confocal images of 6/7 third instar larval NMJs immunolabled with α-HRP (magenta) and α-Brp (A, green), α-CSP (B, green), or α-Syt (C, green). Top panels show merged images. Quantification of relative fluorescence is shown in the right subpanels. Scale bar = 5 µm. (TIF) [file pone.0113494.s002.tif]

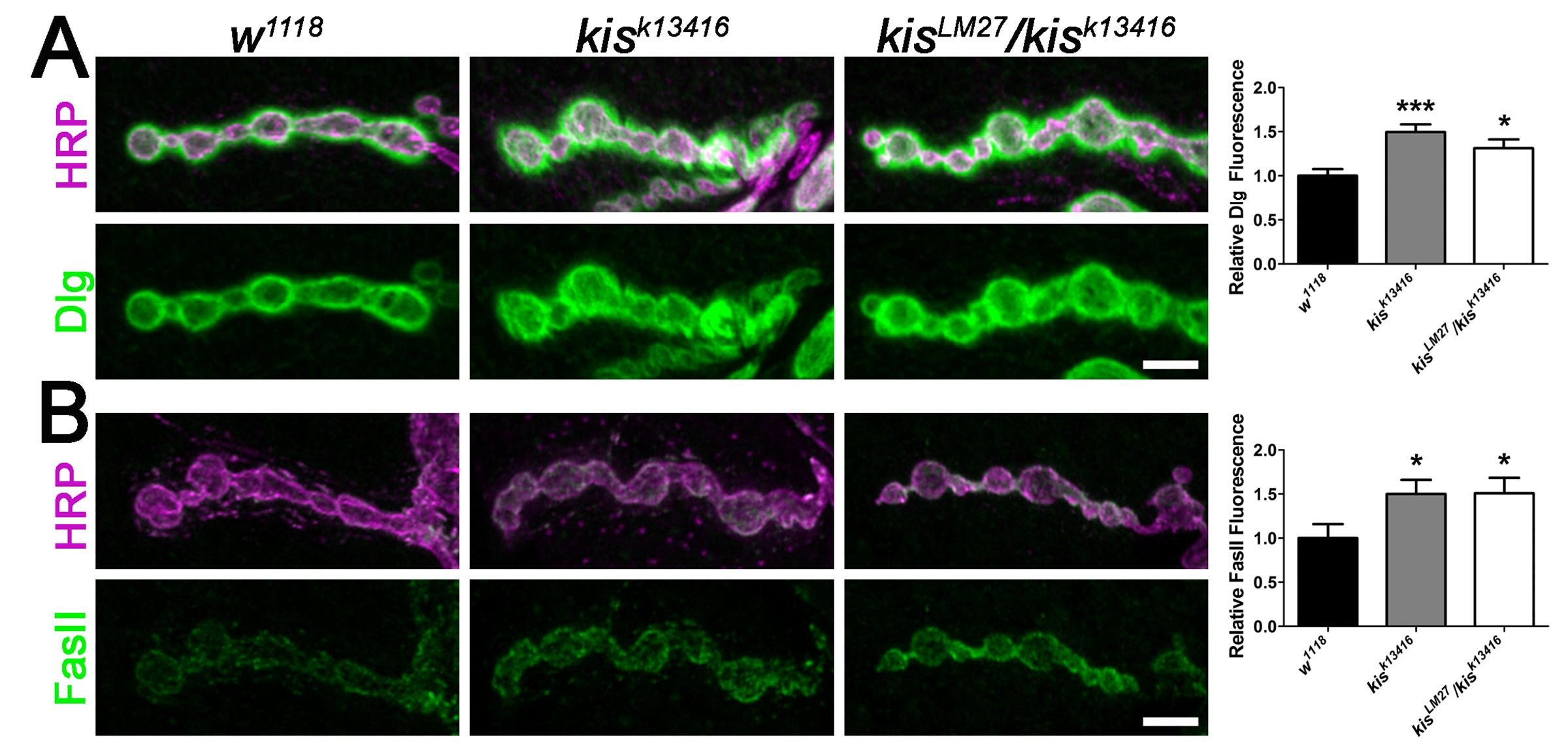

Supplement: Figure S3 — Kis negatively influences synaptic Dlg and FasII levels. (A) High resolution confocal micrographs 6/7 NMJs from third instar larvae immunolabeled with α-HRP (magenta) and α-Dlg (green). Right histogram shows quantification of mean relative Dlg levels in genotypes listed. (B) Confocal images of third instar larval NMJs, muscles 6 and 7, immunolabeled with α-HRP (magenta) and α-FasII (green). Right histogram shows quantification of mean relative Dlg fluorescence in genotypes listed. Scale bar = 5 µm. (TIF) [file pone.0113494.s003.tif]

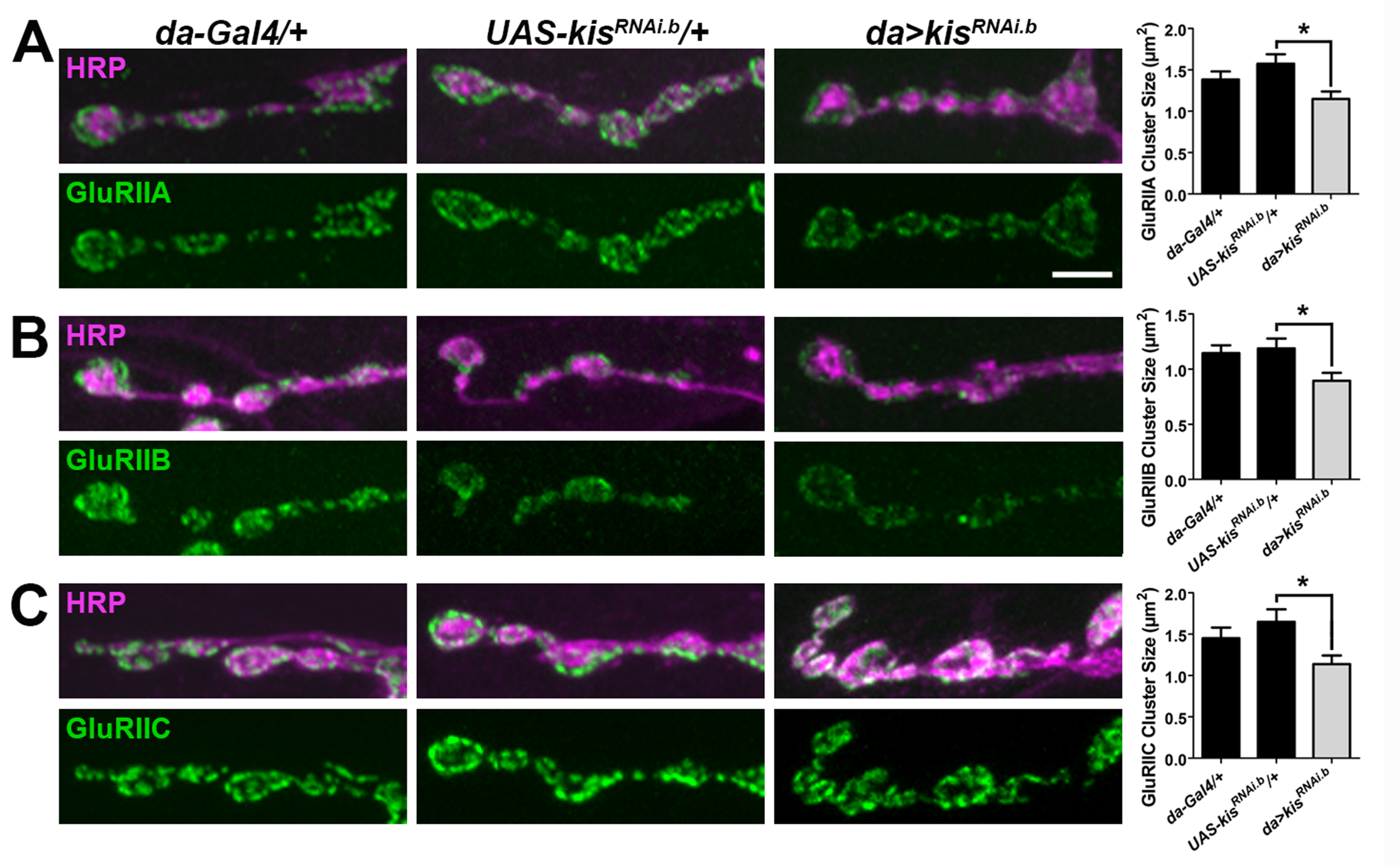

Supplement: Figure S4 — Ubiquitous knockdown of Kis regulates postsynaptic glutamate receptor localization. (A) High resolution confocal images of third instar larval muscles 6 and 7 NMJs immunolabeled with α-HRP (magenta) and α-GluRIIA (green). Quantification of GluRIIA cluster size in µm2 shown in right histogram. (B) Confocal micrographs of third instar larval muscles 6 and 7 NMJs immunolabeled with α-HRP (magenta) and α-GluRIIB (green). Right histogram shows quantification of GluRIIB cluster size in µm2. (C) Confocal images of third instar larval muscles 6 and 7 NMJs immunolabeled with α-HRP (magenta) and α-GluRIIC (green). Quantification of GluRIIC cluster size in µm2 shown in right histogram. Scale bar = 5 µm. (TIF) [file pone.0113494.s004.tif]

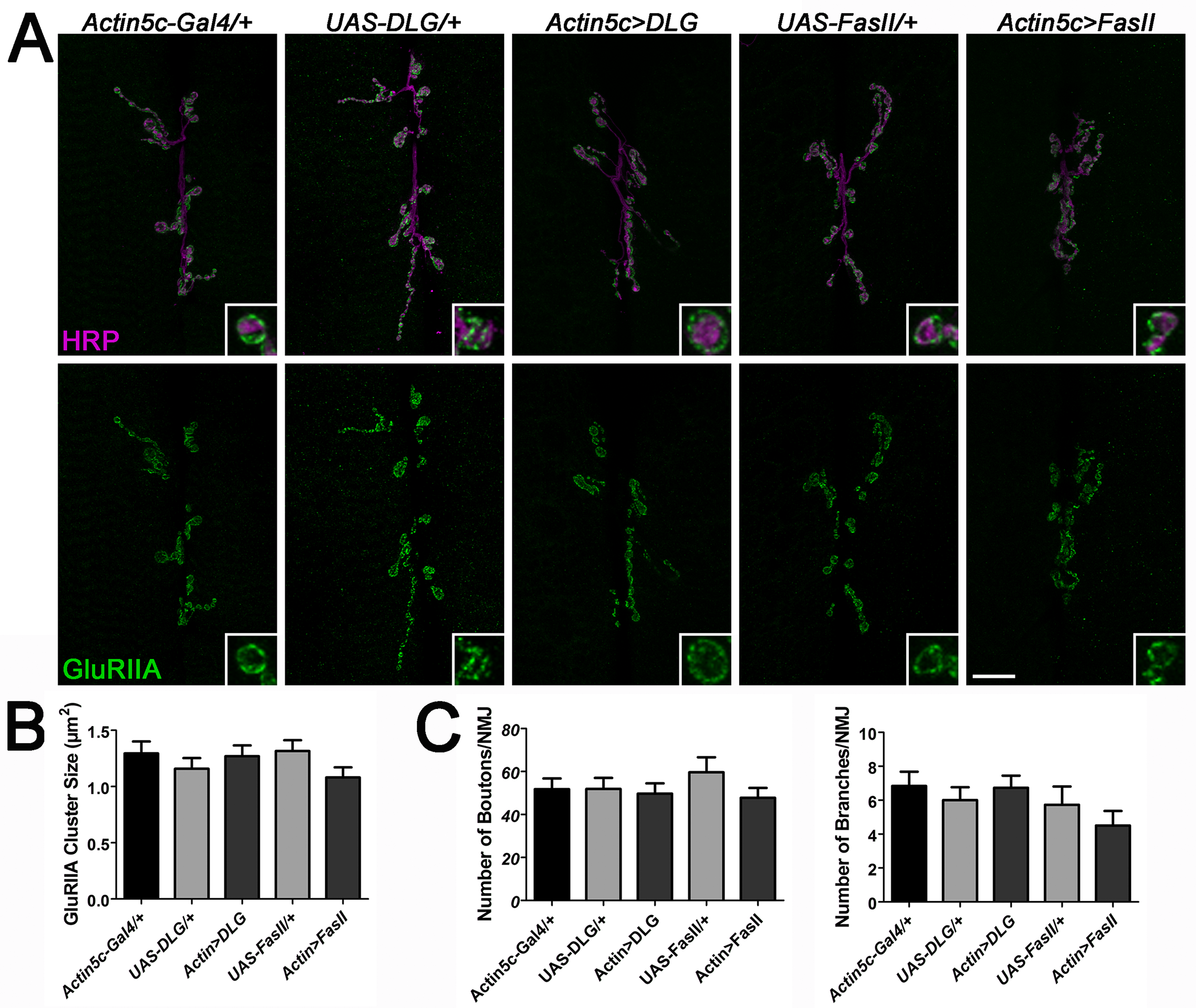

Supplement: Figure S5 — Ubiquitous overexpression of Dlg and FasII does not alter GluR localization. (A) Confocal images of third instar larval 6/7 NMJs immunolabeled with α-HRP (magenta) to label presynaptic motor neurons and α-GluRIIA (green). Dlg and FasII were overexpressed ubiquitously using the Actin5c-Gal4 driver. Insets show high magnification image of a single terminal bouton. Scale bar = 20 µm. (B) Quantification of GluRIIA cluster size in µm2. (C) Quantification of the number of 6/7 NMJ boutons (left) and branches (right) in the genotypes listed. (TIF) [file pone.0113494.s005.tif]
