# Supplementary material for: Kismet Positively Regulates Glutamate Receptor Localization and Synaptic Transmission at the Drosophila Neuromuscular Junction
Source: PLoS One. 2014 Nov 20;9(11):e113494. doi: 10.1371/journal.pone.0113494 (PMC4239079; doi:10.1371/journal.pone.0113494)
Supplement: Table S2 — Selected Gene Ontology clusters related to nervous system function mis-regulated in response to kismet knockdown. (DOCX) [file pone.0113494.s007.docx]

**Supplemental Table 12. Selected Gene Ontology clusters related to nervous system function mis-regulated in response to *kismet* knockdown**

**Biological Process Number of targets affected (down or up)**

Synapse 21 down

Neurotransmitter receptor binding 18 down 15 up

Neuron Projection 8 down

Cognition 54 down 91 up

Neurogenesis 17 down

Learning 12 up
