# Supplementary material for: Kismet Positively Regulates Glutamate Receptor Localization and Synaptic Transmission at the Drosophila Neuromuscular Junction
Source: PLoS One. 2014 Nov 20;9(11):e113494. doi: 10.1371/journal.pone.0113494 (PMC4239079; doi:10.1371/journal.pone.0113494)
Supplement: Table S3 — Synapse target genes showing decreased expression. (DOCX) [file pone.0113494.s008.docx]

**Supplemental Table 3. Synapse target genes decreased**

**Affymetrix ID Gene Name**

1627410_at Amphiphysin

1632216_at Calcineurin A1

1640534_s_at Calcium/calmodulin-dependent protein kinase II

1627821_s_at Dmel_CG2381

1635684_a_at Dmel_CG2999

1638777_at, 1632528_at Dmel_CG33547

1626545_at, 1636377_at Dmel_CG33989

1630846_at Dmel_CG42333

1641131_at Dmel_CG5621

1626101_at Dmel_CG9195

1626739_s_at Glutamate receptor IIC

1635162_at Glutamate receptor IIB

1634465_a_at Glutamate-gated chloride channel

1625838_at Rab-protein 3

1630169_a_at Synapse-associated protein 47kD

1636378_a_at Synapsin

1632620_at Vesicular glutamate transporter

1623999_at dystrophin; snoRNA:122

1629258_at nicotinic Acetylcholine Receptor alpha 7E

1640832_at nicotinic Acetylcholine Receptor alpha 80B

1627219_at, 1637321_at nicotinic Acetylcholine Receptor beta 64B
