# Supplementary material for: Kismet Positively Regulates Glutamate Receptor Localization and Synaptic Transmission at the Drosophila Neuromuscular Junction
Source: PLoS One. 2014 Nov 20;9(11):e113494. doi: 10.1371/journal.pone.0113494 (PMC4239079; doi:10.1371/journal.pone.0113494)
Supplement: Table S4 — Neurotransmitter receptor genes showing decreased expression. (DOCX) [file pone.0113494.s009.docx]

**Supplemental Table 4. Neurotransmitter Receptor genes decreased**

**Affymetrix ID Gene Name**

1629186_a_at Allatostatin Receptor

1639961_at Dmel_CG12370

1632424_at Dmel_CG13995

1625956_at Dmel_CG32547

1637355_at, 1631364_at Dmel_CG33696

1626545_at, 1636377_at Dmel_CG33989

1638336_at Dmel_CG8422

1624469_at Dromyosuppressin receptor 1

1624049_at Dromyosuppressin receptor 2

1634465_a_at Glutamate-gated chloride channel

1626739_s_at Glutamate receptor IIC

1635162_at Glutamate receptor IIB

1635442_a_at Gonadotropin-releasing hormone receptor

1626875_at PDF receptor

1634888_at Sex peptide receptor

1629258_at nicotinic Acetylcholine Receptor alpha 7E

1640832_at nicotinic Acetylcholine Receptor alpha 80B

1627219_at, 1637321_at nicotinic Acetylcholine Receptor beta 64B
