# Supplementary material for: Kismet Positively Regulates Glutamate Receptor Localization and Synaptic Transmission at the Drosophila Neuromuscular Junction
Source: PLoS One. 2014 Nov 20;9(11):e113494. doi: 10.1371/journal.pone.0113494 (PMC4239079; doi:10.1371/journal.pone.0113494)
Supplement: Table S5 — Neurotransmitter receptor genes showing increased expression. (DOCX) [file pone.0113494.s010.docx]

**Supplemental Table 5. Neurotransmitter Receptor genes increased**

**Affymetrix ID Gene Name**

1625101_at Cardioacceleratory peptide receptor

1638649_at Dmel_CG11340

1629280_at Dmel_CG12344

1628222_at Dmel_CG30106

1626472_at Dmel_CG30340

1635672_at Dmel_CG34411

1639301_at Dmel_CG4395

1628250_at Dmel_CG5911

1637003_at Dmel_CG6927

1629962_at Dmel_CG8916

1630762_at Dmel_CG9918

1623309_at Neuropeptide Y receptor-like

1637028_at SIFamide receptor

1626723_at Tachykinin-like receptor at 86C

1634889_at, 1624643_a_at allatostatin C receptor 2
